# Physical and chemical impacts of a major storm on a temperate lake: a taste of things to come?

**DOI:** 10.1007/s10584-018-2302-3

**Published:** 2018-10-03

**Authors:** R. Iestyn Woolway, John H. Simpson, David Spiby, Heidrun Feuchtmayr, Ben Powell, Stephen C. Maberly

**Affiliations:** 1 Department of Meteorology, University of Reading, Reading, UK; 2 School of Ocean Sciences, Bangor University, Menai Bridge, Anglesey, UK; 3 Environment Agency, Penrith, UK; 4 Centre for Ecology & Hydrology, Lancaster, UK

## Abstract

Extreme weather can have a substantial influence on lakes and is expected to become more frequent with climate change. We explored the influence of one particular extreme event, Storm Ophelia, on the physical and chemical environment of England’s largest lake, Windermere. We found that the substantial influence of Ophelia on meteorological conditions at Windermere, in particular wind speed, resulted in a 25-fold increase (relative to the study-period average) in the wind energy flux at the lake-air interface. Following Ophelia, there was a short-lived mixing event in which the Schmidt stability decreased by over 100 Jm^−2^ and the thermocline deepened by over 10 m during a 12-h period. As a result of changes to the strength of stratification, Ophelia also changed the internal seiche regime of Windermere with the dominant seiche period increasing from ~ 17 h pre-storm to ~ 21 h post-storm. Following Ophelia, there was an upwelling of cold and low-oxygenated waters at the southern-end of the lake. This had a substantial influence on the main outflow of Windermere, the River Leven, where dissolved oxygen concentrations decreased by ~ 48%, from 9.3 to 4.8 mg L^−1^, while at the mid-lake monitoring station in Windermere, it decreased by only ~ 3%. This study illustrates that the response of a lake to extreme weather can cause important effects downstream, the influence of which may not be evident at the lake surface. To understand the impact of future extreme events fully, the whole lake and downstream-river system need to be studied together.

**Electronic supplementary material:**

The online version of this article (10.1007/s10584-018-2302-3) contains supplementary material, which is available to authorized users.

## Introduction

Extreme climatic events, such as storms, high winds, floods and heat waves, can have a major influence on aquatic ecosystems (Robson and Hamilton 2003; Jankowski et al. 2006; Tsai et al. 2008; Jöhnk et al. 2008; Giling et al. 2017; Kasprzak et al. 2017; Ji et al. 2018). There is evidence that the frequency and severity of extreme events are increasing as a result of directional climate change (Coumou and Rahmstorf 2012; Hansen et al. 2012), and there is a growing realisation that predicting the effects of future climatic conditions on aquatic ecosystems must explicitly incorporate extreme events, superimposed upon the long-term climate trends. Understanding the impact of extreme weather is important because of the negative effects they can have on ecosystem services that lakes provide, such as the provision of safe water for drinking and irrigation, recreational use, supporting biodiversity and economic benefits such as fisheries and tourism (Wagner and Adrian 2009; Klug et al. 2012; de Eyto et al. 2016; Michalak 2016).

Severe storms are a major type of extreme event and can have large effects on lakes. Storms influence lakes primarily by loading of terrestrial material with catchment runoff as a result of heavy precipitation (Riis and Sand-Jensen 1998; de Eyto et al. 2016; Zwart et al. 2016) and mixing of the water column by high wind stress (Klug et al. 2012), which along with surface heating/cooling is one of the most important factors driving physical processes within lakes (Wüest and Lorke 2003). In particular, wind stress can act to induce oscillatory internal wave motions (seiches), which are observed widely to be the most energetic large-scale motions in stratified lakes and are responsible for driving turbulence and, thus, mixing (Imberger 1998). By disrupting the vertical thermal structure and mixing regime of lakes, storms can have a major influence on the ecosystem (Giling et al. 2017; Kasprzak et al. 2017). These pulsed disturbances have been shown to have a substantial influence on, among other things, community structure (Jones et al. 2008; Beaver et al. 2013), nutrient concentrations (Robarts et al. 1998), lake metabolism (Giling et al. 2017) and carbon dioxide emissions from lakes (Jones et al. 2009). A detailed understanding of the impact of extreme weather on lake ecosystems is therefore essential for climate change impact and water management studies (Zhu et al. 2014; Michalak 2016).

Extreme storms are expected to become more frequent and intense with climate change (Beniston et al. 2007; Rockel and Woth 2007; Gastineau and Soden 2009), although with considerable regional variability (IPCC 2013). Some lakes already experience frequent extreme weather events, to which, they may be well adapted (Jones et al. 2008, 2009) and thus future changes in storm intensity may have little impact. However, the occurrence of more intense and frequent storms in regions where they are currently uncommon, such as the United Kingdom, could result in substantial changes in lake ecosystem structure and functioning. Climate change scenarios predict more frequent and heavier future storms in Western Europe (Hov et al. 2013; Haarsma et al. 2013), and thus a potential increase in the occurrence of extreme weather in the United Kingdom as a result of, among other things, warmer sea surface temperatures (Baatsen et al. 2015). There is some evidence that this may indeed be gradually taking place. For example, in October 2017, the extratropical Storm Ophelia (hereafter Ophelia) reached the offshore western coast of the British Isles producing severe thunderstorms, flooding events, power outages and gusts of 90 mph in some regions (UK Met Office 2017). By the time Ophelia made landfall, it was re-classified as a ‘post-tropical Storm’, but just a few hours earlier, it was still a Category 3 hurricane. Ophelia was described as ‘unusual’ as it had the force and effect of a hurricane without the title but also because of its easterly location in this part of the Atlantic Ocean. It was later confirmed as the easternmost Category 3 hurricane ever recorded (UK Met Office 2017).

To begin investigating the impact of severe storms on freshwater ecosystems in the United Kingdom, we studied the effects of Ophelia on Windermere, the largest natural lake in England. We predicted that Ophelia would have an influence on stratification and mixing dynamics in Windermere and we were interested to see, if occurred, these changes were sufficient to influence the internal seiche regime and result in the upwelling of low-oxygenated bottom waters to the lake surface as well as the main outflow of Windermere, the River Leven.

## Materials and methods

### Study site and observations

**Fig. 1:**
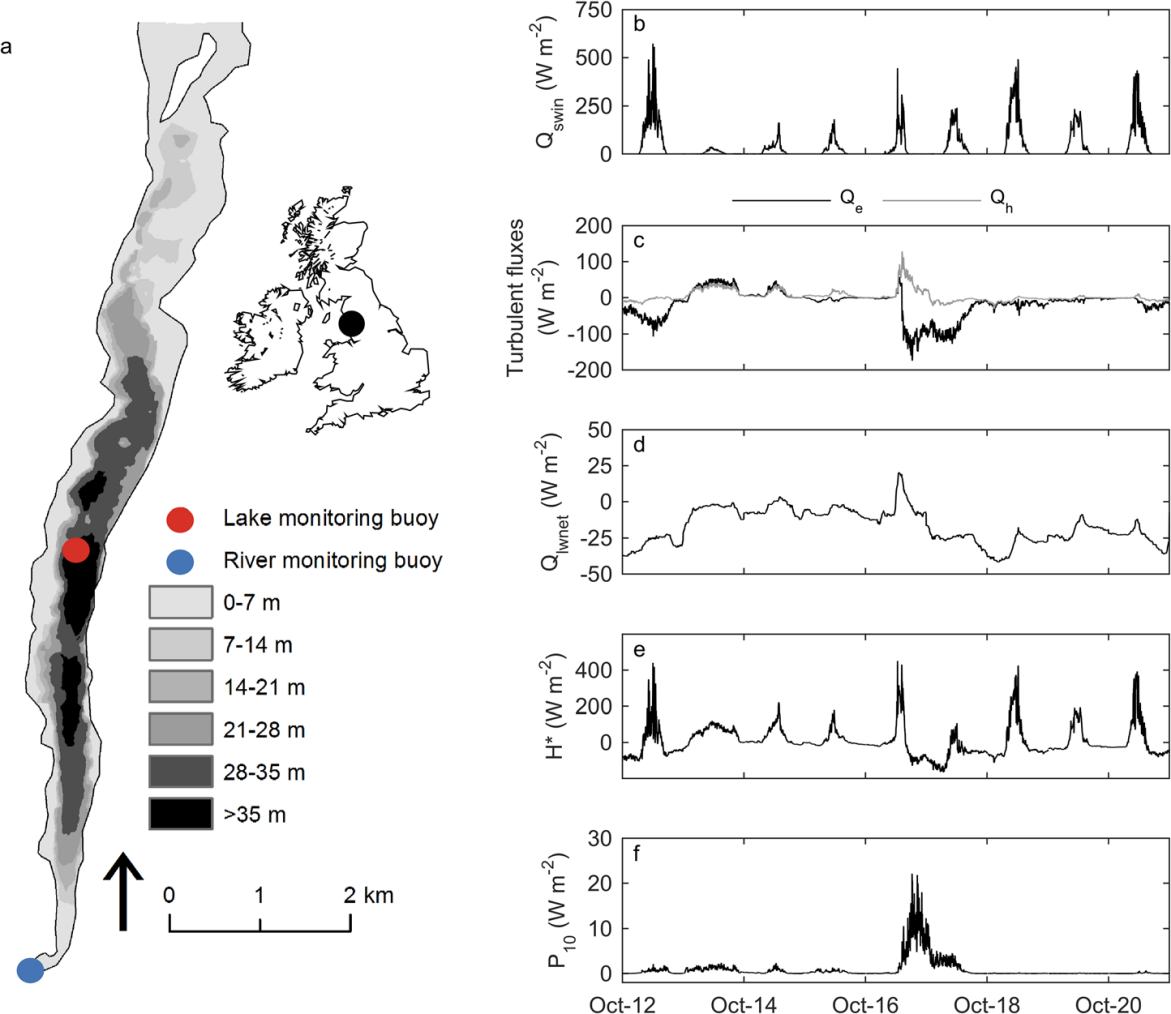
**a** Bathymetric map of the south basin of Windermere (Miller et al. 2014), showing the location of the lake (red) and river (blue) monitoring stations, and calculated **b** net incoming solar radiation (*Q*_*swin*_), **c** latent (*Q*_*e*_, black) and sensible (*Q*_*h*_, grey) heat fluxes, **d** net thermal (i.e. longwave) radiation (*Q*_*lwnet*_ = *Q*_*lwin*_ - *Q*_*lwout*_) and **e** effective heat flux (H_*_). Each of the surface heat fluxes is positive when the direction of heat transfer is from the atmosphere to the lake (i.e. acting to heat the lake surface). Also shown is **f** the wind energy flux (*P*_*10*_). The arrow in Fig. 1a indicates north. The inset shows the location of the study region in the United Kingdom

Our study is based on measurements in the south basin of Windermere (Fig. 1a, English Lake District; 54.343°N, − 2.941°E). The south basin of Windermere, which is separated from the north basin by a shallow (2 m) sill, is long (~ 10 km) and narrow (width, < 1 km) with a surface area of ~ 6.7 km^2^, a maximum depth of 42 m and a mean depth of 16.8 m. The observations reported here from Windermere covered the period October 12th to October 21st 2017, which is from 4 days before and 5 days after Ophelia (October 16th, 2017).

Water temperature profiles in Windermere were recorded at the deepest point of the lake with 12 stainless-steel sheathed platinum resistance thermometers. The sensors were at depths of 1, 2, 4, 7, 10, 13, 16, 19, 22, 25, 30 and 35 m below the lake surface. Meteorological conditions above the lake were also recorded, including air temperature (*T*, K) and relative humidity (*RH*, %), measured 2.3 m above the lake surface; wind speed (*u*_*z*_, m s^−1^) and direction, measured 2.7 m above the lake surface; as well as incoming solar radiation (*Q*_*sw*_, W m^−2^) and air pressure (*p*, mbar). High-resolution surface (within the upper 1 m) dissolved oxygen concentrations were measured by a Hydrolab DS5X sonde. These observations were recorded at 4-min intervals using a Campbell Scientific CR1000 data logger. On October 3rd and 31st 2017, Secchi depth and profiles of dissolved oxygen and temperature were measured manually with a Secchi disc and a Yellow Springs Instruments ProODO sensor, respectively.

Water motion was measured at the centre of the south basin from a bottom-mounted Acoustic Doppler Current Profiler (ADCP) (Teledyne RDI Workhorse 600 kHz ADCP) that recorded average profiles at intervals of ∆t= 60 s based on 50 sub-pings. These were averaged spatially to yield the components of horizontal velocity with a root mean square (rms) uncertainty of ~ 1 cm s^−1^ and with a vertical bin size of ∆z= 1 m. Water column velocities were converted in this study to axial and transverse components by rotating coordinates by 9° clockwise (Simpson et al. 2015; Woolway and Simpson 2017), the orientation of the lake from North (Fig. 1).

The data from Windermere were supplemented by high-frequency (30-min) measurements on the River Leven, at the weir at Newby Bridge that forms the outflow of Windermere, about 4.8 km downstream of the monitoring buoy (Fig. 1a). The high-frequency data, recorded with a YSI EXO2 sonde, include observations of surface water temperature, dissolved oxygen, pH and specific conductivity. Also, flow measurements were made near the north shore of the weir, approximately 200 m downstream of the main impoundment. Rainfall observations from four sites (Coppermines, 54.37°N–3.08°E; High Newton, 54.24°N–2.92 °E; Oxen Park, 54.28°N–3.05°E; Blackmoss, 54.38°N–2.88°E) within the English Lake District were also used in this study. These data were provided by the Environment Agency.

To compare wind speed observations during Ophelia with long-term average conditions, we analysed wind speed data from a nearby meteorological station available from HadISD (Dunn et al. 2012), which is a quality-controlled synoptic meteorological dataset used for climate applications at sub-daily resolution. Specifically, data from a meteorological station situated approximately 24 km from the lake (station ID: 032250-99999; 54.5**°**N, − 2.7**°**E) was used. As wind speed can vary substantially at seasonal timescales (Woolway et al. 2017), we only analyse wind speed observations during October, when comparing with those observed in Windermere during Ophelia.

### Analysis

The effective heat flux, H_*_ (W m^−2^), which can be used to determine whether the surface layer of a lake is gaining or losing heat (MacIntyre et al. 2002), was calculated following Kim (1976) as:

1 $$ {H}_{\ast }={Q}_s-{R}_0\left[\left(2-2\exp \left(-{z}_{mix}\times {K}_d\right)\right)/\left({z}_{mix}\times {K}_d-\exp \left(-{z}_{mix}\times {K}_d\right)\right)\right] $$

where *Q*_*s*_ is the net surface energy flux (W m^−2^; see below), *R*_*0*_ is the photosynthetically active radiation (in W m^−2^), calculated as 42% of the total solar radiation (*Q*_*swin*_, W m^−2^) (Woolway et al. 2015a) and *K*_*d*_ = 0.54 m^−1^ is the light attenuation coefficient, calculated as a function of Secchi depth (= 1.75/Secchi depth) (Woolway et al. 2015a). The algorithms of Read et al. (2011) were used to calculate the depth of the upper mixed layer, *z*_*mix*_ (m), as well as the depth of the thermocline.

The net surface energy flux, *Q*_*s*_, was calculated as:

2 $$ {Q}_s={Q}_{swin}+{Q}_{lwin}-{Q}_{lwout}+{Q}_h+{Q}_e, $$

where *Q*_*swin*_ was estimated as *Q*_*swin*_ = (1 - *α*_*sw*_)*Q*_*sw*_ where *α*_*sw*_ is the shortwave albedo, estimated from Fresnel’s Equation (Woolway et al. 2015b). *Q*_*lwin*_ is the incoming thermal radiation (i.e. longwave; W m^−2^), estimated based on the emissivity and temperature of the atmosphere following Crawford and Duchon (1999), using the algorithms of Woolway et al. (2015b). We assumed that 3% of thermal radiation was reflected at the lake surface (Brutsaert 1982). Emitted longwave radiation, *Q*_*lwout*_ (W m^−2^), was estimated as *Q*_*lwout*_ = 0.97*σT*_*o*_^4^, where *σ* is the Stefan-Boltzmann constant (= 5.67 × 10^−8^ W m^−2^ K^−4^), and *T*_*o*_ is the surface water temperature (K).

*Q*_*h*_ and *Q*_*e*_ are the sensible (Eq. 3) and latent (Eq. 4) heat fluxes, respectively, positive when heat flux is from the atmosphere to the lake surface (W m^−2^), estimated with bulk aerodynamic methods:

3 $$ {Q}_h={\rho}_{\mathrm{a}}{C}_{pa}{C}_h{u}_z\left(T-{T}_o\right), $$

4 $$ {Q}_e={\uprho}_{\mathrm{a}}{\mathrm{L}}_{\mathrm{V}}{\mathrm{C}}_{\mathrm{e}}{\mathrm{u}}_{\mathrm{z}}\left({\mathrm{q}}_{\mathrm{z}}-{\mathrm{q}}_{\mathrm{s}}\right), $$

where *ρ*_*a*_ is the air density (kg m^−3^), estimated as a function of air pressure, air temperature and humidity (Chow et al. 1988; Verburg and Antenucci 2010), *C*_*pa*_ = 1005 J kg^−1^ K^−1^ is the specific heat of dry air at constant pressure, *L*_*v*_ is the latent heat of vaporisation (J kg^−1^), *C*_*h*_ and *C*_*e*_ are the turbulent transfer coefficients for heat and humidity, respectively, which were adjusted for measurement height, wind speed and atmospheric stability (Zeng et al. 1998) by applying stability functions (Woolway et al. 2015b), *q*_*s*_ is the specific humidity at saturation (kg kg^−1^) and *q*_*z*_ is the specific humidity (kg kg^−1^) calculated from relative humidity, air temperature and air pressure.

As a reference for the rate of energy input to a lake from the atmosphere, we use *P*_*10*_ the rate of working in a horizontal plane above the lake surface (Lombardo and Gregg 1989):

5 $$ {P}_{10}={C}_d{\rho}_a\overline{{u_{10}}^3}, $$

where *C*_*d*_ is the transfer coefficient for momentum which, similar to *C*_*h*_ and *C*_*e*_, was adjusted for atmospheric stability using the algorithms of Woolway et al. (2015b). *u*_*10*_ is the wind speed adjusted to a height of 10 m above the lake surface, calculated by accounting for atmospheric stability and measurement height (Woolway et al. 2015b).

Indices used to describe lake mixing and stratification, Schmidt stability and Lake Number, were computed using the algorithms of Read et al. (2011). Schmidt stability (Idso 1973) describes the resistance to mechanical mixing caused by the potential energy inherent in stratification: it is near-zero when the lake is mixed and increases as stratification strengthens. The Lake Number describes the degree of thermocline tilting as a function of stratification, wind forcing and basin morphometry (Imberger and Patterson 1990). Bathymetry data used in this study for calculating the Lake Number were from Ramsbottom (1976). A Lake Number of greater than 1 suggests that stratification is strong and dominates the forces introduced by surface wind energy, while for a Lake Number less than 1, stratification is weak with respect to wind stress and the thermocline is expected to experience strong tilting and, in turn, the lake will likely experience upwelling of hypolimnetic waters. When calculating the Lake Number, we used a low-pass filter with a cut-off frequency corresponding to ¼ of the internal seiche period to reduce observational noise (MacIntyre et al. 2009).

To estimate the periods of the internal seiche modes in Windermere, we followed the methods used by Simpson et al. (2011). Specifically, the periods and modal structure of the internal seiche motions were investigated by cross-spectral analysis of the ADCP time series. In particular, the cross-spectrum between the along-lake velocity at each level (i.e. bins) and that at the lowest bin level were computed. The resulting spectra were compiled into depth-frequency plots of the cross-spectral energy between different levels. Forming the co-spectrum of the velocities at each level with the near-bed flow has the advantage of improving the signal to noise ratio because the near-bed flow tends to be dominated by seiche motions and is relatively free from ‘noise’ associated with less regular motions further up the water column. We applied the cross-spectral analysis technique to 16-day periods of ADCP data before and after Ophelia.

## Results

Ophelia had a substantial influence on meteorological conditions at Windermere, in particular surface air temperature and wind speed (Fig. S1). In contrast, there was little or no rainfall during Ophelia (Fig. S2a), as also indicated by the general reduction in flow at Newby Bridge over the study period (Fig. S2b). Maximum air temperature and wind speed during October 16–17 were 6 °C and 14 m s^−1^ higher than the mean conditions observed throughout the study period. In addition, the maximum wind speed observed during October 16–17 (~ 19.0 m s^−1^) was over four times greater than the average wind speed observed from a nearby meteorological station during the same time of year from 1979 to 2017 (~ 4.1 m s^−1^). Relative humidity decreased as a result of the change in air temperature, resulting in a substantial difference in the surface energy fluxes (Fig. 1). In particular, the exchange of turbulent energy at the air-water interface (i.e. latent and sensible heat fluxes) differed considerably during October 16–17 (Fig. 1c). The sensible heat flux (*Q*_*h*_) increased to a maximum of approximately 120 W m^−2^ whereas the latent heat flux (*Q*_*e*_) increased (i.e. negative heat flux) to a maximum of approximately − 170 W m^−2^. The increase in *Q*_*h*_ was a result of the increase in wind speed and the air-water temperature difference (see Eq. 3), the latter a result of the increase in air temperature and decrease in surface water temperature (as a result of the increase in wind mixing energy, see below) at this time (Fig. S3). The decrease in *Q*_*e*_ was caused by the increase in wind speed and a decrease in the air-water humidity difference, the latter being caused by the increase in air temperature resulting in a decrease in humidity above the lake surface (Fig. S3). Net longwave radiation, *Q*_*lwnet*_ = *Q*_*lwin*_ - *Q*_*lwout*_, was typically negative (mean ≈− 17 ± 12 W m^−2^) throughout the study period, meaning that *Q*_*lwnet*_ was generally acting to cool the lake surface, and that *Q*_*lwout*_ (mean ≈ 370 ± 1 W m^−2^) was, on average, greater than *Q*_*lwin*_ (mean ≈ 353 ± 13 W m^−2^). However, during October 16–17, *Q*_*lwnet*_ increased to ~ 20 W m^−2^, following closely the increase in air temperature, to which *Q*_*lwin*_ is closely related (Fig. 1d).

**Fig. 2:**
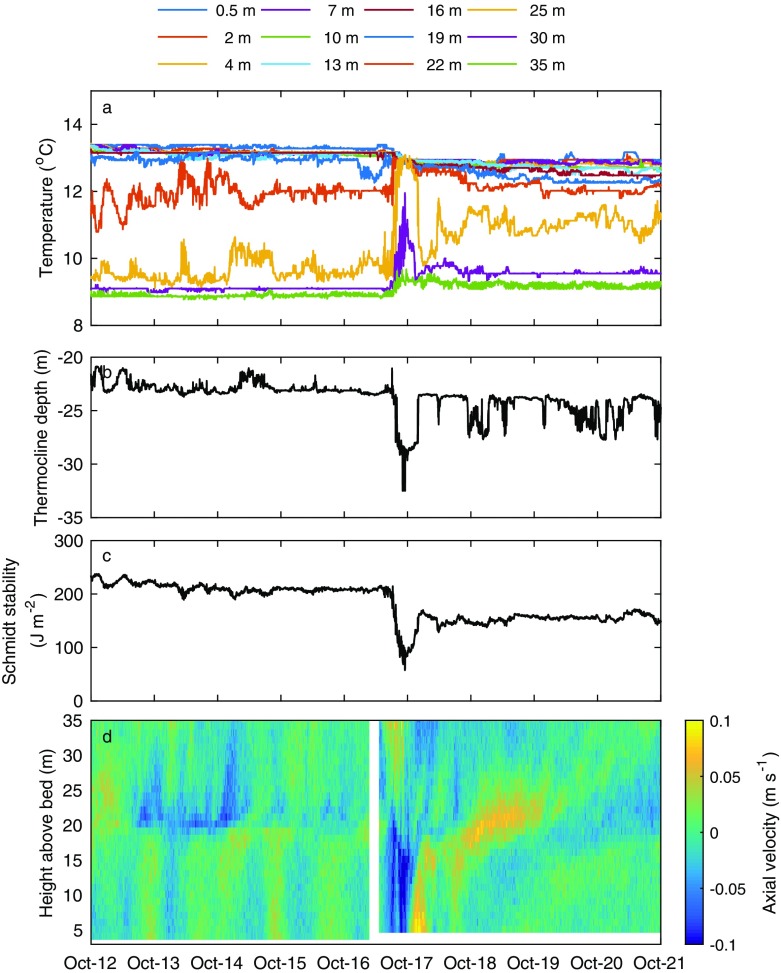
**a** Depth-resolved lake temperature, where the individual lines represent temperatures measured by different thermistors, the depth of which are shown in the legend. **b** Depth of the thermocline, calculated using the algorithms of Read et al. (2011). **c** Calculated Schmidt stability (Read et al. 2011). **d** Axial component of water column velocities measured at 1 m intervals; positive values indicate water flowing north, negative values indicate water flowing south

Accounting for all of the surface energy fluxes acting on the surface layer, we estimated the effective heat flux H_*_ (see Eq. 1). During October 16–17, there was a negative H_*_, indicating net cooling of the surface layer of Windermere (Fig. 1e), following closely the increase in negative *Q*_*e*_ (Fig. 1c). At this time, there was also a large increase in the mechanical energy flux, *P*_*10*_, due to wind stress, with *P*_*10*_ increasing to a maximum of 22 W m^−2^ following Ophelia against an average of 0.85 W m^−2^ observed during the study period (Fig. 1f), an increase by a factor of 25. Enhanced surface cooling and an increase in wind energy during Ophelia resulted in a short-lived mixing event in Windermere (Fig. 2a) and a rapid deepening of the thermocline (Fig. 2b). Specifically, the depth of the thermocline deepened by 10 m in a 12-h period from an average of ~ 23 m prior to Ophelia to a maximum of ~ 33 m during October 16–17. There was a corresponding sudden drop in lake thermal stability (Fig. 2c). After the storm, the Schmidt stability (≈ 144 J m^−2^) was over 60 J m^−2^ (~ 25%) lower than that observed before Ophelia.

**Fig. 3:**
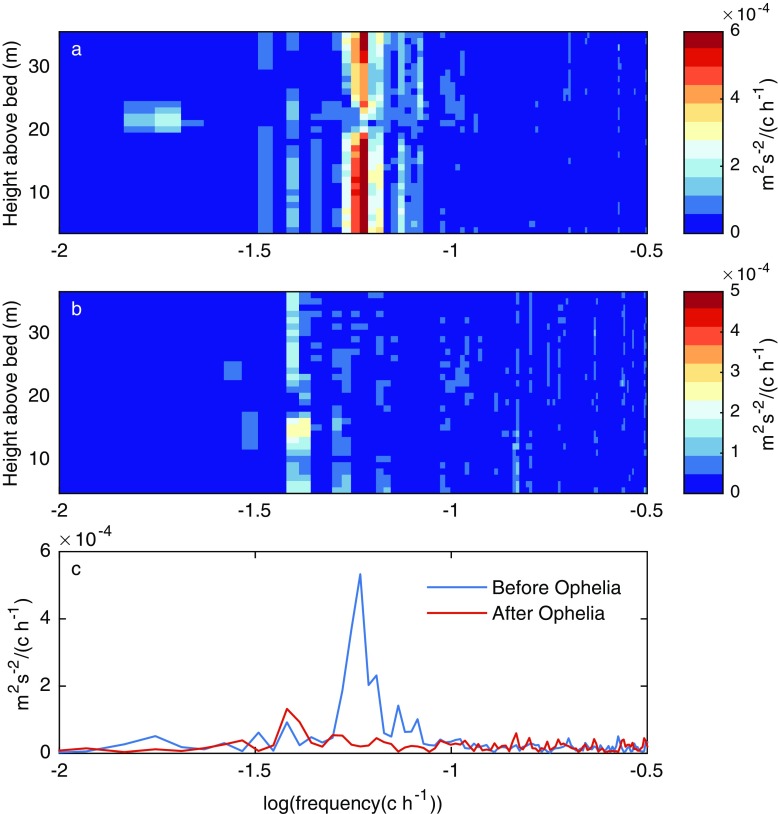
Axial velocity cross-spectrum for 16 days before and after Storm Ophelia. Plots shown are the amplitude of the cross-spectral power density between the axial velocity at each level (i.e. ADCP bin) and the near-bed velocity for **a** before (day of year 273–289) and **b** after (day of year 291–307) the storm. Also shown is **c** a comparison of the depth-averaged amplitude of the cross-spectral power density before and after Ophelia

The increase in wind speed and the subsequent change in the strength of stratification following Ophelia also resulted in a radical change in the axial component of the water column velocities (Fig. 2d). Specifically, during October 16–17, there was a large negative axial flow (i.e. towards the southern end of the lake) of ~ 0.1 m s^−1^ within the hypolimnion of Windermere. This was accompanied by a large positive axial flow (i.e. towards the northern end of the lake) of ~ 0.1 m s^−1^ within the epilimnion (Fig. 2d). In Windermere, well-defined oscillations in the water column were observed throughout the study period and current velocities were generally highly structured in the vertical (Fig. 2d). Prior to Ophelia, surface and bottom water velocities in Windermere were generally in antiphase, which is characteristic of first mode internal seiche activity (Fig. 2d). Application of the cross-spectral analysis technique (see Methods) to the water column velocity data before Ophelia indicates a narrow and well-defined band of energy corresponding to a period of ~ 17 h (Fig. 3a). At this frequency, there is a drop in spectral energy at ~ 23 m, corresponding to the depth of the metalimnion (within which lies the thermocline). This indicates clearly that the spectral peak corresponded to the first vertical seiching mode. There is no evidence of spectral peaks at frequencies higher than that of the first vertical mode prior to the impact of Ophelia. The cross-spectral method was then applied to the water column velocities measured after Ophelia (Fig. 3b). There are clear differences in the spectral characteristics of the axial velocities after Ophelia compared to those computed before the storm. Specifically, there was a marked change in the seiching period of Windermere with much longer period motions (~ 21 h) observed (Fig. 3b). Comparison of the spectral averages before and after the storm also demonstrates a considerable reduction in seiche energy (Fig. 3c), likely a result of the wind speed prior to Ophelia (~ 4.4 m s^−1^) being higher than observed after the storm (~ 3.1 m s^−1^). There was little difference in the period of wind forcing before and after Ophelia (not shown).

**Fig. 4:**
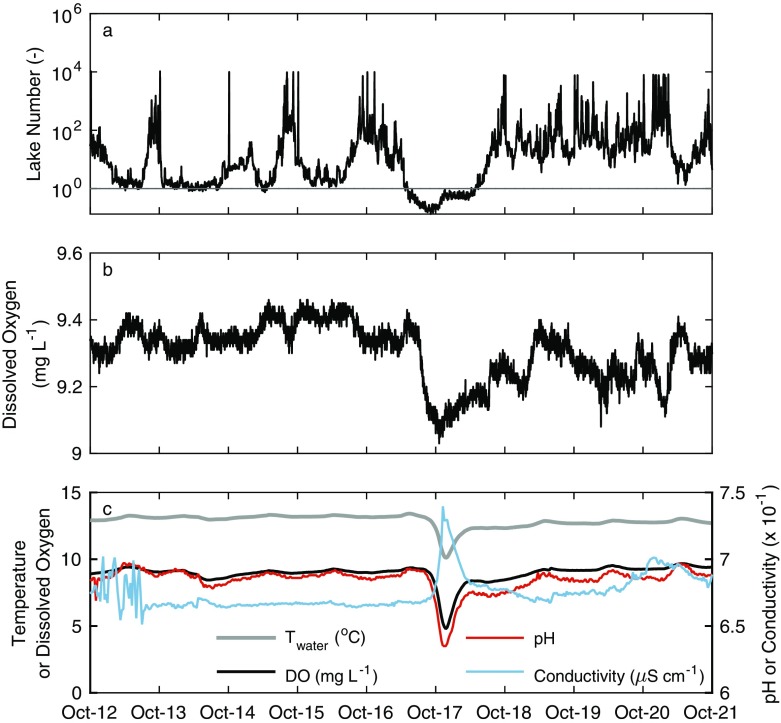
**a** Lake Number, calculated using the algorithms of Read et al. (2011), with the horizontal line representing a Lake Number of 1. Lower values would be indicative of conditions favouring an upwelling event. Also shown are high-resolution observations of **b** surface dissolved oxygen from the surface of Windermere (4-min temporal resolution) and **c** surface water temperature (grey), dissolved oxygen (black), pH (red) and specific conductivity (blue) measured in the River Leven (30-min temporal resolution)

An upwelling event in Windermere is suggested from the Lake Number, which decreased to below 1 on October 16–17 (Fig. 4a). This interpretation also agrees with the observed southward axial flow within the hypolimnion, as identified from the ADCP data (e.g. Fig. 2d). While the oxygen concentration in the surface layer of Windermere at the centre of the south basin of the lake only changed from 9.4 to 9.1 mg L^−1^ (~ 3% decrease) (Fig. 4b), exposure of the hypolimnion to the lake surface resulted in an input of cold, low-oxygenated water (Fig. S4) at the southern-end of the lake which can be observed in the high-resolution data measured at the River Leven (Fig. 4c). On October 17, water temperature decreased by ~ 3 °C to a minimum of 10.1 °C, dissolved oxygen decreased by > 4 mg L^−1^ from 9.3 to 4.8 mg L^−1^ (~ 48% decrease), and pH decreased by ~ 0.6 to a minimum of 6.35 while specific conductivity increased from about 66.7 to 74.0 μS cm^−1^. Peak excursions occurred within 90 min of each other. The low oxygen excursion lasted for about 15 h, but only fell below 7 mg L^−1^ (about 64% air saturation) for 6 h and below 5 mg L^−1^ (about 44% air saturation) for about 1.5 h. After the effect of Ophelia had passed, there were reductions in temperature of 0.72 °C, oxygen of 0.2 mg L^−1^ and pH of 0.08 and an increase in conductivity of 6.4 μS cm^−1^. The peak excursions in the River Leven lagged behind the mixing event in Windermere by about 4 h.

## Discussion

We investigated the influence of Storm Ophelia on the physical and chemical environment of Windermere, the largest natural lake in England, as well as the outflowing river, the Leven. Windermere is one of the best studied lakes in the world and has been the focus of studies in physical limnology and climate change impacts on lakes for well-over 60 years (Mortimer 1952; Talling 1999; Maberly and Elliott 2012). However, the response of Windermere, as well as other lakes in the United Kingdom, to extreme weather has been largely unexplored, primarily since extreme events in this part of the world have not occurred frequently in the past. In addition, while previous studies have investigated the influence of extreme events on the physical environment of some lakes in other regions (Klug et al. 2012; Jennings et al. 2012), these have focused primarily on within-lake thermal metrics (such as stability and mixing depths) but, unlike our study in Windermere, have not investigated the implications of within-lake processes on downstream conditions. Furthermore, in this study, an ADCP was used in conjunction with a meteorological station to investigate the influence of extreme weather on internal seiche motions and energy fluxes at the lake-air interface, which has not previously been explored, but is essential for understanding how atmospheric forcing and extreme weather can affect the lake-river ecosystem.

Based on high-frequency observational data, we found that Ophelia had a substantial influence on meteorological conditions at Windermere. One of the most noticeable effects of Ophelia was the increase in wind speed and the subsequent increase in the wind energy flux, *P*_*10*_, which increased to magnitudes that were 25 times greater (~ 22 W m^−2^) than the average during the study period and considerably greater than the maximum *P*_*10*_ (~ 6 W m^−2^) observed by Woolway and Simpson (2017) during a 3-month period in Windermere in 2013. *P*_*10*_ is an important metric for understanding the influence of atmospheric forcing on a lake ecosystem (Ravens et al. 2000; Wüest et al. 2000; Rueda and Schladow 2009; Bouffard et al. 2012) and it is related closely with the rate of working of the wind, which can be used to quantify the energy input to surface waters. In particular, during periods of high *P*_*10*_, the rate of working by the wind, as well as the kinetic energy of the flow, also tends to be high, resulting in enhanced dissipation of turbulent kinetic energy and vertical mixing (Woolway and Simpson 2017).

The increase in wind speed at Windermere during Ophelia had a substantial influence on the turbulent energy fluxes at the lake-air interface, such as the evaporative heat flux (i.e. the latent heat flux). In particular, the increase in wind speed, to which the latent heat flux is closely related (see Eq. 4), changed markedly. Latent heat loss at the lake-air interface was found to increase as a result of Ophelia, not only because of the increase in wind speed but also because of the decrease in the lake-air humidity difference (i.e. the difference between the saturated humidity at the lake surface temperature minus the observed humidity immediately above the lake surface). The latter was caused by above-normal air temperatures at Windermere, as a result of the southerly airflow drawing warm air from lower latitudes during the storm, and the subsequent decrease in humidity above the lake surface. The latent heat flux, which is typically the dominant turbulent heat loss process occurring in lakes (Woolway et al. 2018), is important for the lake ecosystem as it influences, among other things, the thermal structure. In particular, some of the most important physical effects of climate change on the physics, chemistry and biology of lakes are associated with changes in thermal structure, heat budgets and ultimately the fluxes of heat and energy at the lake-air interface (McCormick 1990; Livingstone 2003; Fink et al. 2014; Schmid et al. 2014).

Ophelia had a marked influence on the vertical temperature structure in Windermere with the thermocline deepening rapidly as a result of the storm. The changes in thermal stratification had a marked influence on internal seiching. Previous studies have shown that the modal periods of internal seiches correlate closely with the evolution of stratification, with longer period motions occurring when stratification is weaker (Simpson et al. 2011). This relationship was evident during the impact of Ophelia when the seiching period of Windermere changed abruptly from ~ 17 h pre-storm to ~ 21 h post-storm and that the energy of the internal seiche decreased substantially. The latter was likely a result of higher wind speed prior to Ophelia. One might also expect an increase in seiching energy as a result of changes in the wind forcing period (Woolway and Simpson 2017). Previous studies have shown that the efficiency of energy transfer from the wind to the lake is higher when the internal seiche period matches that of the wind period (Woolway and Simpson 2017), but this was not evident during this study.

Future climate change scenarios predict that maximum wind speeds over Europe will become stronger with climate change, with a general tendency of more frequent and stronger extreme winds by the end of the century (Leckebusch et al. 2006; Beniston et al. 2007; Rockel and Woth 2007). Specifically, there is evidence that hurricane-force storms reaching Europe will be enhanced in the future as a result of climate change. In particular, modelling studies demonstrate that an increase in tropical sea surface temperatures with climatic warming will extend north-eastwards the breeding ground of tropical cyclones, resulting in an extension of the hurricane genesis area and, in turn, more intense tropical cyclones reaching Europe (Zhao and Held 2012; Murakami et al. 2012; Haarsma et al. 2013). In addition, a warmer future climate could result in the re-intensification of ex-hurricanes as they approach Europe, increasing the chances of extreme winds and also expand north-eastwards the impact region of such storms (Baatsen et al. 2015). There is evidence of an increase in the number of hurricanes that turned north-east, similar to Ophelia, from 1948 to 2014 (Feser et al. 2015).

Previous studies have investigated the influence of extreme weather on lake thermal stability and have shown that a physical disturbance can last from several hours to days or weeks (Jennings et al. 2012; Klug et al. 2012; Giling et al. 2017). While the storm-induced mixing event in Windermere was short-lived, previous studies have demonstrated that pulsed disturbances of this kind can induce strong and protracted impacts on the lake ecosystem (Giling et al. 2017). In particular, studies have shown that while the physical structure of a lake can recover fully within a few days following a storm, biogeochemical processes can take substantially longer to return to pre-storm levels.

During the study period, there was minimal change in dissolved oxygen (~ 3% decrease) at the mid-lake monitoring station in the south basin of Windermere. However, dissolved oxygen concentrations decreased greatly (~ 48% decrease) at the surface of River Leven, situated at the southern end of the lake. Our interpretation is that the decrease in dissolved oxygen in the River Leven on October 17 was imported through entrainment of water from the hypolimnion of Windermere. The lower temperature, pH, O_2_ and higher conductivity all indicate input of water from depth. The Lake Number, which has been used previously to estimate the flux of oxygen across the thermocline in lakes (Robertson and Imberger 1994), also indicated high potential for increased diapycnal mixing and the occurrence of an upwelling event (i.e. Lake Number < 1) as a result of Ophelia.

The conditions at the bottom of relatively deep productive lakes, such as Windermere, are very different to those at the surface. The temperature is lower, oxygen concentrations are often low or zero and concentrations of nutrients and CO_2_ are often high. Stratified lakes allow a spatial ‘escape’ for motile organisms able to avoid unfavourable conditions (Clegg et al. 2007), while in rivers, chemical conditions are virtually homogenous with depth and spatial escape is of limited possibility. Furthermore, the build-up of, for example, low oxygen concentration at depth in a lake occurs gradually over many weeks and is essentially a ‘press’ or ‘ramp’ disturbance (Lake 2000): that is a disturbance that persists over a period of time and either reaches a constant level or increases in intensity. The long-term ecological effects of exposure to these types of disturbance are known in terms of the effects of low oxygen on fish distribution, fitness and survival of organisms such as fish at low oxygen (Roberts et al. 2009). In contrast, the effect of Ophelia on the River Leven was essentially a sudden, short-term ‘pulse’ disturbance, involving changes to several different environmental variables. Some studies have suggested that extreme weather events could be just as important as gradual trends for the long-term trajectories of ecosystems (Perga et al. 2018), but there is very little known about the possible consequences of this type of disturbance, and more research is clearly needed given that pulse disturbances are likely to increase in frequency as extreme weather events become more common.

## Electronic supplementary material

ESM 1 (DOCX 7787 kb)

## References

[CR1] Baatsen M, Haarsma RJ, Van Delden AJ, de Vries H (2015). Severe autumn storms in future Western Europe with a warmer Atlantic Ocean. Clim Dyn.

[CR2] Beaver J, Casamatta D, East T (2013). Extreme weather events influence the phytoplankton community structure in a large lowland subtropical lake (Lake Okeechobee, Florida, USA). Hydrobiologia.

[CR3] Beniston M, Stephenson DB, Christensen OB (2007). Future extreme events in European climate: an exploration of regional model projections. Clim Change.

[CR4] Bouffard D, Boegman L, Rao YR (2012). Poincaré wave-induced mixing in a large lake. Limnol Oceanogr.

[CR5] Brutsaert WH (1982). Evaporation into the atmosphere: theory, history, and applications.

[CR6] Chow VT, Maidment DR, Mays LW (1988). Applied hydrology.

[CR7] Clegg MR, Maberly SC, Jones RI (2007). Behavioural response as a predictor of seasonal depth distribution and vertical niche separation in freshwater phytoplanktonic flagellates. Limnol Oceanogr.

[CR8] Coumou D, Rahmstorf S (2012). A decade of weather extremes. Nat Clim Change.

[CR9] Crawford TM, Duchon CE (1999). An improved parameterization for estimating effective emissivity for use in calculating daytime downwelling longwave radiation. J Appl Meteorol.

[CR10] de Eyto E, Jennings E, Ryder E (2016). Response of a humic lake ecosystem to an extreme precipitation event: physical, chemical, and biological implications. Inland Waters.

[CR11] Dunn RJH, Willett KM, Thorne PW (2012). HadISD: a quality-controlled global synoptic report database for selected variables at long-term stations from 1973-2011. Clim Past.

[CR12] Feser F, Schubert-Frisius M, von Storch H (2015). Hurricane Gonzalo and its extratropical transition to a strong European storm. Bull Am Meteorol Soc.

[CR13] Fink G, Schmid M, Wahl B, Wolf T, Wüest A (2014). Heat flux modifications related to climate-induced warming of large European lakes. Water Resour Res.

[CR14] Gastineau G, Soden BJ (2009). Model projected changes of extreme wind events in response to global warming. Geophys Res Lett.

[CR15] Giling DP, Nejstgaard JC, Berger SA (2017). Thermocline deepening boosts ecosystem metabolism: evidence from a large-scale lake enclosure experiment simulating a summer storm. Glob Chang Biol.

[CR16] Haarsma RJ, Hazeleger W, Severijns C (2013). More hurricanes to hit western Europe due to global warming. Geophys Res Lett.

[CR17] Hansen J, Sato M, Ruedy R (2012). Perception of climate change. Proc Natl Acad Sci U S A.

[CR18] Hov Ø, Cubasch U, Fischer E (2013). Extreme weather events in Europe: preparing for climate change adaptation.

[CR19] Idso SB (1973). On the concept of lake stability. Limnol Oceanogr.

[CR20] Imberger J (1998) Flux paths in a stratified lake: a review. In: Imberger J (ed) Physical processes in lakes and oceans, Coastal and Estuarine Studies. AGU, pp 1–18

[CR21] Imberger J, Patterson JC (1990). Physical limnology. Adv Appl Mech.

[CR22] Stocker TF, Qin D, Plattner G-K, Tignor M, Allen SK, Boschung J, Nauels A, Xia Y, Bex V, Midgley PM, IPCC (2013). Climate change 2013: the physical science basis. Contribution of Working Group I to the Fifth Assessment Report of the Intergovernmental Panel on Climate Change.

[CR23] Jankowski T, Livingstone DM, Bührer H (2006). Consequence of the 2003 European heat wave for lake temperature profiles, thermal stability, and hypolimnetic oxygen depletion: implications for a warmer world. Limnol Oceanogr.

[CR24] Jennings E, Jones S, Arvola L (2012). Effects of weather-related episodic events in lakes: an analysis based on high-frequency data. Freshw Biol.

[CR25] Ji G, Havens KE, Beaver JR, East TL (2018). Recovery of plankton from hurricane impacts in a large shallow lake. Freshw Biol.

[CR26] Jöhnk KD, Huisman J, Sharples J (2008). Summer heatwaves promote blooms of harmful cyanobacteria. Glob Chang Biol.

[CR27] Jones SE, Chiu CY, Kratz TK (2008). Typhoons initiate predictable change in aquatic bacterial communities. Limnol Oceanogr.

[CR28] Jones SE, Kratz TK, Chiu CY, McMahon KD (2009). The influence of typhoons on annual CO2 flux from a sub-tropical humic lake. Glob Chang Biol.

[CR29] Kasprzak P, Shatwell T, Gessner MO (2017). Extreme weather event triggers cascade towards extreme turbidity in a clear-water lake. Ecosystems.

[CR30] Kim JW (1976). Generalized bulk model of oceanic mixed layer. J Phys Oceanogr.

[CR31] Klug JL, Richardson DC, Ewing HA (2012). Ecosystem effects of a tropical cyclone on a network of lakes in northeastern North America. Environ Sci Technol.

[CR32] Lake PS (2000). Disturbance, patchiness, and diversity in streams. J N Am Benthol Soc.

[CR33] Leckebusch GC, Koffi B, Ulbrich U (2006). Analysis of frequency and intensity of winter storm events in Europe on synoptic and regional scales from a multi-model perspective. Clim Res.

[CR34] Livingstone DM (2003). Impact of secular climate change on the thermal structure of a large temperate central European lake. Clim Change.

[CR35] Lombardo CP, Gregg MC (1989). Similarity scaling of viscous and thermal dissipation in a convecting surface boundary layer. J Geophys Res-Oceans.

[CR36] Maberly SC, Elliott JA (2012). Insights from long-term studies in the Windermere catchment: external stressors, internal interactions and the structure and function of lake ecosystems. Freshw Biol.

[CR37] MacIntyre S, Romero JR, Kling GW (2002). Spatial-temporal variability in surface layer deepening and lateral advection in an embayment of Lake Victoria, East Africa. Limnol Oceanogr.

[CR38] MacIntyre S, Fram JP, Kushner PJ (2009). Climate-related variations in mixing dynamics in an Alaskan arctic lake. Limnol Oceanogr.

[CR39] McCormick MJ (1990). Potential changes in thermal structure and cycle of Lake Michigan due to global warming. Trans Am Fish Soc.

[CR40] Michalak AM (2016). Study role of climate change in extreme threats to water quality. Nature.

[CR41] Miller H, Bull J, Cotterill CJ, Dix J (2014) Windermere multibeam bathymetry data. University of Southampton, UK 10.5258/SOTON/364801

[CR42] Mortimer CH (1952). Water movements in lakes during summer stratification: evidence from the distribution of temperature in Windermere. Philos Trans R Soc Lond.

[CR43] Murakami H, Wang Y, Yoshimura H (2012). Future changes in tropical cyclone activity projected by the new high-resolution MRI-AGCM. J Clim.

[CR44] Perga Marie-Elodie, Bruel Rosalie, Rodriguez Laura, Guénand Yann, Bouffard Damien (2018). Storm impacts on alpine lakes: Antecedent weather conditions matter more than the event intensity. Global Change Biology.

[CR45] Ramsbottom AE (1976) Depth charts of the Cumbrian lakes. Freshwater Biological Association

[CR46] Ravens TM, Kocsis O, Wüest A, Granin N (2000). Small-scale turbulence and vertical mixing in Lake Baikal. Limnol Oceanogr.

[CR47] Read JS, Hamilton DP, Jones ID (2011). Derivation of lake mixing and stratification indices from high-resolution lake buoy data. Environ Model Softw.

[CR48] Riis T, Sand-Jensen K (1998). Development of vegetation and environmental conditions in an oligotrophic Danish lake over 40 years. Freshw Biol.

[CR49] Robarts RD, Waiser MJ, Hadas O (1998). Relaxation of phosphorus limitation due to typhoon-induced mixing in two morphologically distinct basins of Lake Biwa, Japan. Limnol Oceanogr.

[CR50] Roberts JJ, Ludsin SA, Hoeoek TO (2009). Effects of hypolimnetic hypoxia on foraging and distributions of Lake Erie yellow perch. J Exp Mar Biol Ecol.

[CR51] Robertson DM, Imberger J (1994). Lake number, a quantitative indicator of mixing used to estimate changes in dissolved-oxygen. Int Rev Gesamten Hydrobiol.

[CR52] Robson BJ, Hamilton DP (2003). Summer flow event induces a cyanobacterial bloom in a seasonal Western Australia. Mar Freshw Res.

[CR53] Rockel B, Woth K (2007). Extremes of near-surface wind speed over Europe and their future changes as estimated from an ensemble of RCM simulations. Clim Change.

[CR54] Rueda F, Schladow G (2009). Mixing and stratification in lakes of varying horizontal length scales: scaling arguments and energy partitioning. Limnol Oceanogr.

[CR55] Schmid M, Hunziker S, Wüest A (2014). Lake surface temperatures in a changing climate: a global sensitivity analysis. Clim Change.

[CR56] Simpson JH, Wiles PJ, Lincoln BJ (2011). Internal seiche modes and bottom boundary-layer dissipation in a temperate lake from acoustic measurements. Limnol Oceanogr.

[CR57] Simpson JH, Lucas NS, Powell P, Maberly SC (2015). Dissipation and mixing during the onset of stratification in a temperate lake, Windermere. Limnol Oceanogr.

[CR58] Talling JF (1999) Some English lakes as diverse and active ecosystems: a factual summary and source book. Freshwater Biological Association

[CR59] Tsai J-W, Kratz TK, Hanson PC (2008). Seasonal dynamics, typhoons and the regulation of lake metabolism in a subtropical humic lake. Freshw Biol.

[CR60] United Kingdom Meteorological Office (2017) Ex-hurricane Ophelia report. URL: https://www.metoffice.gov.uk/climate/uk/interesting/2017-ophelia

[CR61] Verburg P, Antenucci JP (2010) Persistent unstable atmospheric boundary layer enhances sensible and latent heat loss in a tropical great lake: Lake Tanganyika. J Geophys Res:115. 10.1029/2009JD012839

[CR62] Wagner C, Adrian R (2009). Cyanobacteria dominance: quantifying the effects of climate change. Limnol Oceanogr.

[CR63] Woolway RI, Simpson JH (2017). Energy input and dissipation in a temperate lake during the spring transition. Ocean Dyn.

[CR64] Woolway RI, Jones ID, Feuchtmayr H, Maberly SC (2015). A comparison of the diel variability in epilimnetic temperature for five lakes in the English Lake District. Inland Waters.

[CR65] Woolway RI, Jones ID, Hamilton DP (2015). Automated calculation of surface energy fluxes with high-frequency lake buoy data. Environ Model Softw.

[CR66] Woolway R. Iestyn, Verburg Piet, Merchant Christopher J., Lenters John D., Hamilton David P., Brookes Justin, Kelly Sean, Hook Simon, Laas Alo, Pierson Don, Rimmer Alon, Rusak James A., Jones Ian D. (2017). Latitude and lake size are important predictors of over-lake atmospheric stability. Geophysical Research Letters.

[CR67] Woolway R. Iestyn, Verburg Piet, Lenters John D., Merchant Christopher J., Hamilton David P., Brookes Justin, de Eyto Elvira, Kelly Sean, Healey Nathan C., Hook Simon, Laas Alo, Pierson Don, Rusak James A., Kuha Jonna, Karjalainen Juha, Kallio Kari, Lepistö Ahti, Jones Ian D. (2018). Geographic and temporal variations in turbulent heat loss from lakes: A global analysis across 45 lakes. Limnology and Oceanography.

[CR68] Wüest A, Lorke A (2003). Small-scale hydrodynamics in lakes. Annu Rev Fluid Mech.

[CR69] Wüest A, Piepke G, Van Senden DV (2000). Turbulent kinetic energy balance as a tool for estimating vertical diffusivity in wind-forced stratified waters. Limnol Oceanogr.

[CR70] Zeng X, Zhao M, Dickinson RE (1998). Intercomparison of bulk aerodynamic algorithms for the computation of sea surface fluxes using TOGA COARE and TAO data. J Clim.

[CR71] Zhao M, Held IM (2012). TC-permitting GCM simulations of hurricane frequency response to sea surface temperature anomalies projected for the late-twenty-first century. J Clim.

[CR72] Zhu M, Paerl HW, Zhu G (2014). The role of tropical cyclones in stimulating cyanobacterial (Microcystis spp.) blooms in hypertrophic Lake Taihu, China. Harmful Algae.

[CR73] Zwart JA, Sebestyen SD, Solomon CT, Jones SE (2016). The influence of hydrologic residence time on lake carbon cycling dynamics following extreme precipitation events. Ecosystems.

