# Supplementary material for: Physical and chemical impacts of a major storm on a temperate lake: a taste of things to come?
Source: Clim Change. 2018 Oct 3;151(2):333–47. doi: 10.1007/s10584-018-2302-3 (PMC6404735; doi:10.1007/s10584-018-2302-3)
Supplement: Supplementary file 1 — (DOCX 7787 kb) [file 10584_2018_2302_MOESM1_ESM.docx]

Supporting information for

**Physical and chemical impacts of a major storm on a temperate lake: A taste of things to come?**

R. Iestyn Woolway^1*^, John H. Simpson^2^, David Spiby^3^, Heidrun Feuchtmayr^4^, Ben Powell^2^, Stephen C. Maberly^4^

1. *Department of Meteorology, University of Reading, Reading, UK*
2. *Bangor University, School of Ocean Sciences, Menai Bridge, Anglesey, UK*
3. *Environment Agency, Penrith, UK*
4. *Centre for Ecology & Hydrology, Lancaster, UK*

*Corresponding author: [riwoolway@gmail.com](mailto:riwoolway@gmail.com)

**Contents of this file**

Figure S1-S4


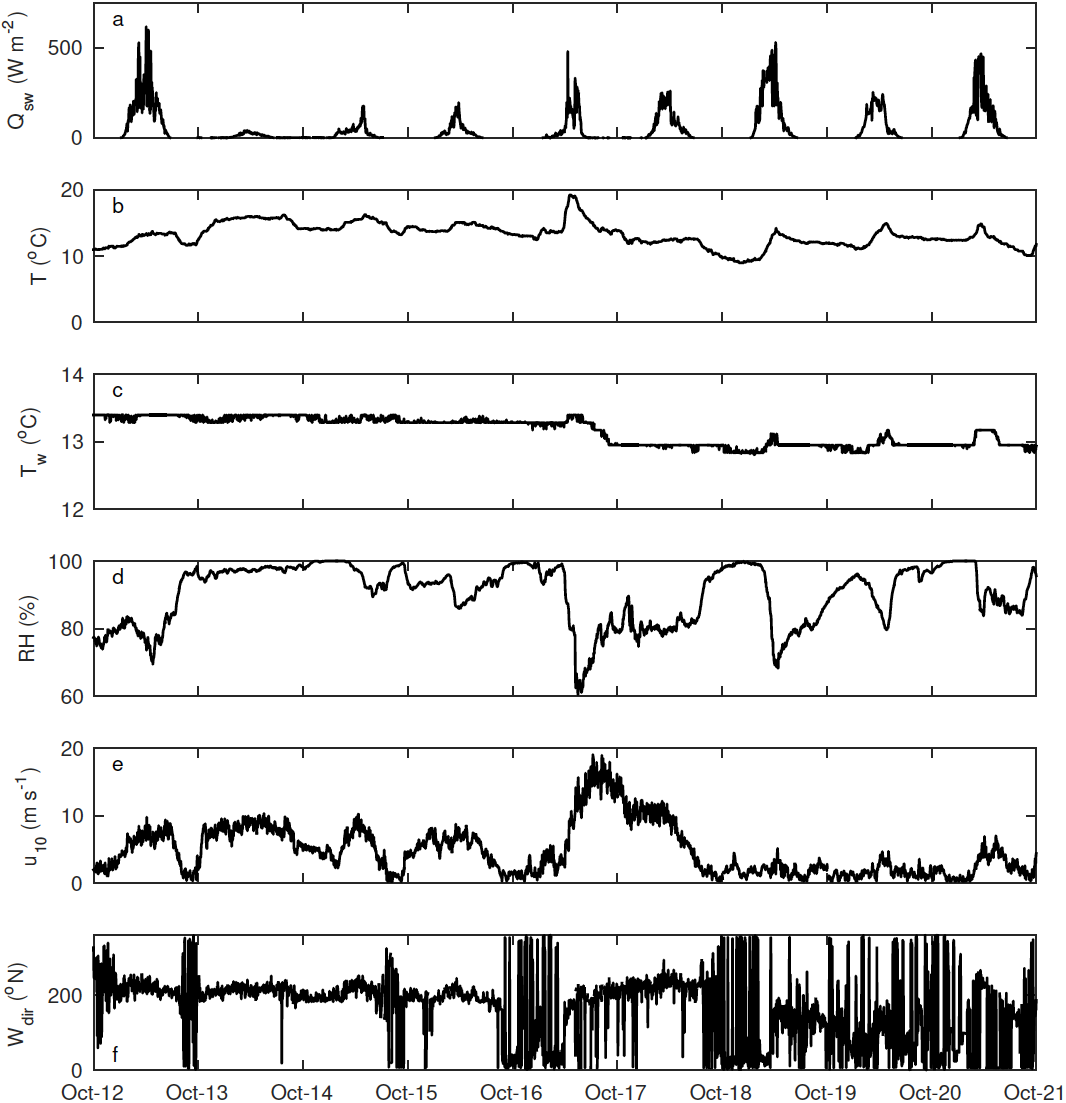


**Figure S1.** High-resolution (4-minute) observational data from the south basin of Windermere showing (a) solar radiation, (b) air temperature measured 2.3 m above the lake surface, (c) surface water temperature measured 1 m below the lake surface, (d) relative humidity measured 2.3 m above the lake surface, (e) surface wind speed adjusted to a height of 10 m (*u_10_*), and (f) wind direction (180° illustrates wind blowing from south to north).

**
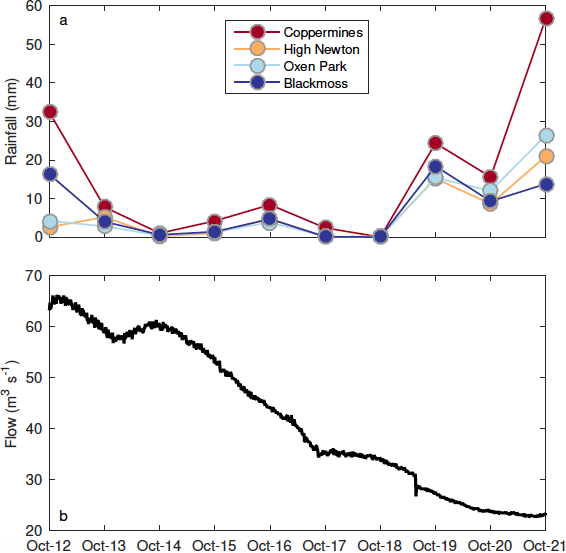
**

**Figure S2.** Observations of (a) daily rainfall at four sites (Coppermines, 54.37 °N -3.08 °E; High Newton, 54.24 °N -2.92 °E; Oxen Park, 54.28 °N -3.05 °E; Blackmoss, 54.38 °N -2.88 °E) in the English Lake District, and (b) high-resolution (30-min) flow rates (m^3^ s^-1^) in the River Leven.


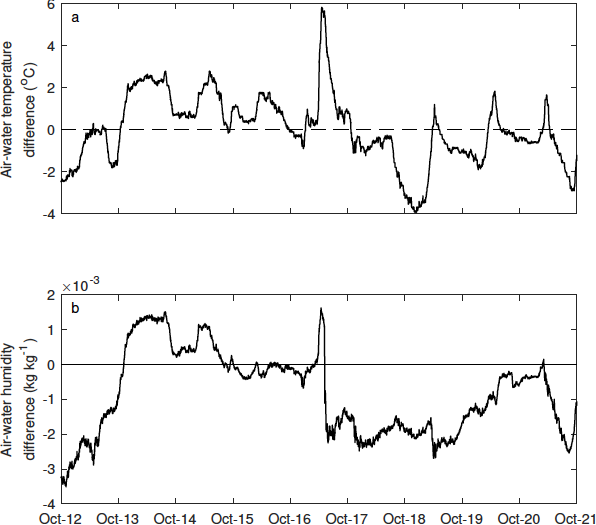


**Figure S3.** Calculated air-water differences of (a) temperature and (b) humidity in the south basin of Windermere during the study period.

**
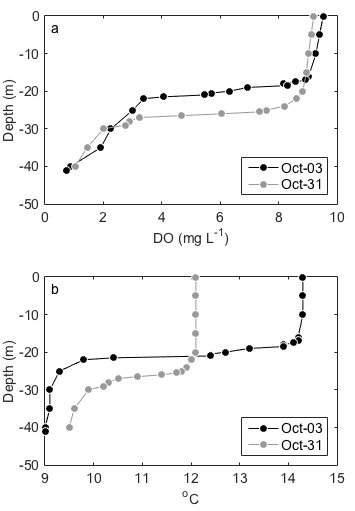
**

**Figure S4.** Observed (a) dissolved oxygen (DO) concentration, and (b) water temperature in the south basin of Windermere before (black) and after (gray) Storm Ophelia.
